# Complete Genome Sequence of Channel Catfish Gastrointestinal Septicemia Isolate *Edwardsiella tarda* C07-087

**DOI:** 10.1128/genomeA.00959-13

**Published:** 2013-11-21

**Authors:** Hasan C. Tekedar, Attila Karsi, Michele L. Williams, Stefanie Vamenta, Michelle M. Banes, Mary Duke, Brian E. Scheffler, Mark L. Lawrence

**Affiliations:** College of Veterinary Medicine, Mississippi State University, Mississippi State, Mississippi, USAa; Food Animal Health Research Program, Ohio State University, Wooster, Ohio, USAb; Genomics and Bioinformatics Research Unit, Agriculture Research Service, United States Department of Agriculture, Stoneville, Mississippi, USAc

## Abstract

*Edwardsiella tarda* is a Gram-negative facultative anaerobe causing disease in animals and humans. Here, we announce the complete genome sequence of the channel catfish isolate *E. tarda* strain C07-87, which was isolated from an outbreak of gastrointestinal septicemia on a commercial catfish farm.

## GENOME ANNOUNCEMENT

*Edwardsiella tarda* is the etiologic agent of acute to chronic edwardsiellosis in fish and other species (1). It is a Gram-negative facultative anaerobe that is motile by peritrichous flagella. Edwardsiellosis is an important fish disease that negatively impacts aquaculture industries throughout the world (2). Moreover, *E. tarda* can cause disease in humans, where it is typically associated with gastrointestinal infections that may progress to hemorrhagic septicemia (3). Historically, *E. tarda* causes a sporadic disease in channel catfish (*Ictalurus punctatus*) called emphysematous putrefactive disease, which may begin as small punctate cutaneous erosions that progress to abscesses deep within the musculature (1). However, *E. tarda* is being increasingly isolated from large outbreaks of gastrointestinal septicemia in commercial channel catfish operations. *E. tarda* isolate C07-087 is from an outbreak of gastrointestinal septicemia in a commercial catfish pond in Mississippi.

To determine the circularized *E. tarda* C07-087 genome sequence, genomic DNA was shotgun sequenced using two different methods: Illumina genome analyzer IIx (8,144,858 reads with 76× coverage) (Illumina, Inc., San Diego, CA) and 454 GS-FLX Titanium platform (312,969 reads with 180× coverage) (Roche Applied Science). CLC Workbench 5.0.1 (CLC bio, Cambridge, MA) and Sequencher 5.1 (Gene Codes Corporation, Ann Arbor, MI) were used to trim sequences, and reads were *de novo* assembled using the CLC Workbench. Unscaffolded gaps were amplified and sequenced by single-primer PCR (4). rRNA gene operons were amplified and sequenced to resolve misassemblies.

The circularized and completed genome of *E. tarda* strain C07-087 was submitted to two different annotation pipelines. The NCBI Prokaryotic Genome Automatic Annotation Pipeline (PGAAP) (5) was used for annotation and submission to GenBank, and RAST annotation (6) was conducted using the Glimmer option to gather detailed information about the *E. tarda* genome.

The *E. tarda* genome consists of one circular chromosome with 3,857,040 bp and 59.6% G+C content. PGAAP annotation predicted 3,525 genes coding for 3,405 proteins. tRNAscan-SE (7) and RNAmmer (8) predicted 95 tRNAs and 8 rRNA operons, respectively. Griffin et al. (9) showed that *E. tarda* consists of two genetic subtypes, DNA groups I and II. Based on sequence comparison and G+C content, C07-087 is in DNA group II. Two *E. tarda* genome sequences from DNA group II were previously reported: that of EIB202, which was isolated from diseased turbot in China (10), and FL6-60, which was isolated from catfish (11).

Plasmid preparations showed that *E. tarda* C07-087 does not carry any plasmids. However, 31,387 bp of the *E. tarda* FL6-60 44,194-bp plasmid is integrated into the *E. tarda* strain C07-087 chromosome, which was confirmed by PCR. The fully sequenced circular *E. tarda* C07-087 genome has unique features relative to previously sequenced *E. tarda* genomes and will be useful for helping to delineate the pathogenesis of gastrointestinal septicemia caused by *E. tarda* in channel catfish.

### Nucleotide sequence accession numbers.

The *E. tarda* strain C07-087 genome was deposited in GenBank under the accession no. CP004141 (GI 469761715). The version described in this paper is version CP004141.1.
